# Ovarian reserve and endothelial health in healthy reproductive-age women

**DOI:** 10.1210/clinem/dgag105

**Published:** 2026-03-12

**Authors:** Ange Wang, Maria E Bleil, Mitchell P Rosen, Rita Redberg, Jue Lin, Dana L Smith, Charles E McCulloch, Marcelle I Cedars

**Affiliations:** Division of Reproductive Endocrinology & Infertility, University of California, San Francisco, CA 94158, USA; Child, Family, & Population Health Nursing, University of Washington, Seattle, WA 98195, USA; Division of Reproductive Endocrinology & Infertility, University of California, San Francisco, CA 94158, USA; Division of Cardiology, Department of Internal Medicine, University of California, San Francisco, CA 94158, USA; Department of Biochemistry and Biophysics, University of California, San Francisco, CA 94158, USA; Department of Biochemistry and Biophysics, University of California, San Francisco, CA 94158, USA; Department of Epidemiology and Biostatistics, University of California, San Francisco, CA 94158, USA; Division of Reproductive Endocrinology & Infertility, University of California, San Francisco, CA 94158, USA

**Keywords:** ovarian aging, antimüllerian hormone, antral follicle count, ovarian reserve, cardiovascular health, endothelial function

## Abstract

**Context:**

While the literature suggests women with diminished ovarian reserve may have increased metabolic risk, implications for long-term health are unknown.

**Objective:**

This work aimed to investigate the relationship between ovarian reserve markers at baseline with a subsequent measure of endothelial dysfunction, as a proxy for cardiovascular risk.

**Methods:**

This prospective cohort study was conducted in a community-based setting. Participants included 322 individuals from the Ovarian Aging Study (OVA), a National Institutes of Health–funded study of ovarian aging (average age 35.4 years at the time of baseline ovarian reserve measurements, and age 45.1 years at the time of endothelial dysfunction measurement). This study investigated the association of ovarian reserve markers at baseline with a subsequent (average of 9.7 years) assessment of cardiovascular risk using the Endothelial and Peripheral Arterial Tone reactive hyperemia index (RHI) score of endothelial function. Secondary outcomes including the American Heart Association PREVENT score, metabolic syndrome, telomere length, and mitochondrial DNA were evaluated.

**Results:**

RHI as a continuous outcome was significantly positively associated both with antimüllerian hormone (AMH) and antral follicle count (AFC) on fully adjusted models (AMH coefficient 0.052; 95% CI, 0.008-0.096; *P* = .02; AFC coefficient 0.017; 95% CI, 0.001-0.032; *P* = .04). For secondary outcomes, the only result that was statistically significant was the association of fully adjusted AFC with metabolic syndrome (odds ratio 0.92; 95% CI, 0.86-0.99; *P* = .02). A sensitivity analysis of the premenopausal cohort (N = 246) had similar findings.

**Conclusion:**

In this longitudinal cohort of women with normal ovarian aging, baseline ovarian reserve markers of AMH and AFC were positively associated with endothelial function as a continuous outcome. Baseline ovarian reserve markers were not related to most secondary outcomes of cellular aging and metabolic risk.

Cardiovascular disease (CVD) is the leading cause of death in women, responsible for approximately one-third of all deaths in women worldwide (1). The relationship between ovarian reserve (total number of eggs remaining) and CV risk has not been well established for individuals with normal ovarian aging (regular menstrual cycles and no premature menopause), particularly in prospective study designs. Previous work suggests women with early diminished ovarian reserve, diagnosed by low oocyte quantity, may have increased CV risk, including measures of coronary atherosclerotic plaque, insulin resistance, C-reactive protein (CRP), and lipid markers (2-4). However, studies are heterogeneous in terms of the outcomes and definition of diminished ovarian reserve (eg, defined as antimüllerian hormone [AMH] < 1.2 ng/ML or antral follicle count [AFC] < 5 (5)). Additionally, it is unclear whether these relationships hold true outside the extremes of premature ovarian insufficiency (POI, defined as menopause prior to age 40 years), though several studies have reported that decreases in AMH are associated with increased risk of CVD, coronary heart disease, and with subclinical atherosclerosis indices in a normal ovarian aging cohort (6, 7). Some studies have also found associations with certain measures of ovarian reserve and markers of cellular aging, which have themselves been linked to CV risk (8-10) though these findings have not been consistently noted across all studies (11), particularly after controlling for confounders (12-16).

Given the importance of sex-specific knowledge of CV risk prediction, this prospective study investigated the association of CV risk, using peripheral endothelial function testing (a noninvasive highly sensitive marker of early CVD risk) (17, 18) and ovarian reserve. Prior studies have not examined baseline ovarian reserve markers in relation to endothelial dysfunction in a longitudinal format. This study used the EndoPAT 200 device (Itamar Medical) to measure endothelial dysfunction (19-21). Reactive hyperemia peripheral arterial tonometry (RH-PAT, as measured by the EndoPAT device) correlates well with measures of coronary and peripheral endothelial function and attenuated RH-PAT indices may identify women at high risk for ischemic heart disease (20, 22-24). Many studies have used EndoPAT to assess microvascular and macrovascular function (22, 25, 26); the Framingham Heart Study exclusively used EndoPAT because of its good reproducibility and ease of testing (24). A study among 140 women scheduled for hospitalization to examine chest pain found RH-PAT was significantly attenuated both in obstructive and nonobstructive CVD compared to nonischemic heart disease (22).

The hypothesis is that ovarian aging is an early marker of CV risk. Understanding this association, and potential underlying mechanisms including markers of cellular aging such as telomere length (TL) and mitochondrial DNA (mtDNA), will improve the ability to identify individuals at risk and potentially yield new insights into early diagnosis and treatment for CVD in women.

## Materials and methods

### Study design and participants

Participants were from the Ovarian Aging Study (OVA), a National Institutes of Health (NIH)-funded study of normal ovarian aging. OVA study and recruitment details have been described previously (27). Briefly, OVA is a multiethnic community-based cohort of women, recruited randomly through the Kaiser of Northern California database, in the San Francisco Bay Area, (recruited in approximately equal proportions of racial/ethnic groups, including Asian [Chinese], Black, Hispanic, and White), with regular ovulatory cycles (every 21-35 days) and no major medical illnesses and reproductive organs intact. Participants self-reported race and ethnicity and confirmed both parents were of the same racial/ethnic groups as declared by the participant. Race and ethnicity were asked to ensure a diverse recruitment across racial/ethnic groups, and enrollment was specifically designed to have equal numbers across racial/ethnic groups as this may affect outcomes of interest (as well as report data on understudied minority groups). Ethnicity and race were queried at the same time, with ethnicity (Hispanic/non-Hispanic) followed by race. Patients who selected Hispanic for ethnicity were classified as Hispanic, otherwise they were classified by race.

**Figure 1:**
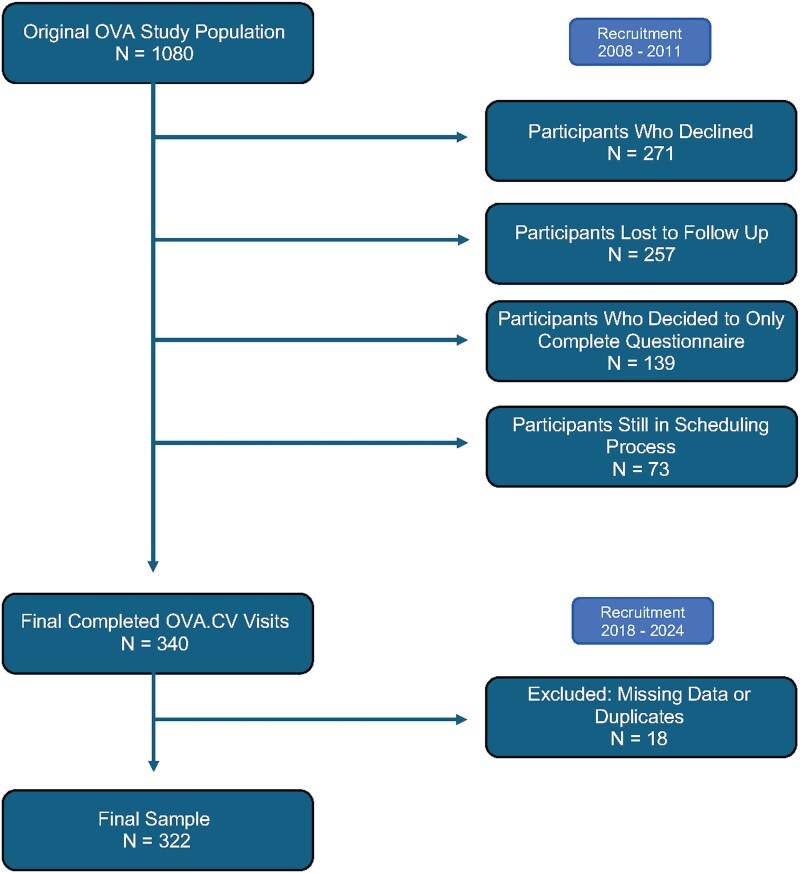
OVA enrollment timeline and final OVA.CV cohort. OVA is the Original Ovarian Aging cohort. OVA.CV is the follow-up cohort assessing cardiovascular risk.

At the time of enrollment, OVA participants were aged 25 to 45 years recruited in equal proportions across 4 5-year age groups. A subset of participants also completed an in-person follow-up visit beginning in 2018 (age 32-52 at the time of the endothelial health measurement). No participants had been on any hormonal medication (oral contraceptive pills, hormone replacement) for at least 2 months. Menopausal status was evaluated. At this visit, baseline measures (including laboratory tests and body measurements and markers of ovarian reserve: AMH/AFC) were repeated and additional CV risk markers were assessed, including endothelial health using EndoPAT (Endothelial and Peripheral Arterial Tone, N = 322 women; Fig. 1). For all visits, participants were off all hormonal medication for at least 2 months and were at least 12 months from pregnancy. Institutional review board approval was obtained through the University of California, San Francisco.

### Outcomes and ascertainment

This was a prospective cohort study investigating the association of ovarian reserve markers (AMH and AFC) at study baseline with a subsequent assessment of CV risk using the EndoPAT score of endothelial dysfunction (conducted an average of 9.7 years after baseline).

For AMH measurement, samples were stored at −80 °C, batched, and analyzed at the Ligand Assay and Analysis Core Laboratory at the University of Virginia (Human ELISA Kit pico-AMH, Ansh Labs LLC; AB_2783675). The lowest amount of AMH in a sample that can be detected with a 95% probability is 1.2 pg/mL or 0.0086 pMol/L. The intra-assay coefficient of variation percentage is 1.8 and the interassay coefficient of variation percentage is 5.4. The AMH samples were batched across participants and within participants—as well as 10% run in duplicate to confirm values and reduce interassay variability. AFC was collected through transvaginal ultrasonography by trained physicians and advanced practice practitioners in a standardized format (28). Ultrasound machines were different at baseline compared to follow-up study; however, 50 participants were scanned simultaneously with both old (Shimadzu SDU-450XL with a variable 4-8 mHz vaginal transducer) and new (General Electric Voluson S8 with an 8-mHz vaginal transducer) machines and showed correlation of more than 96%. Baseline ovarian reserve markers were also studied in relation to additional secondary outcomes including American Heart Association (AHA) PREVENT calculator, metabolic syndrome, and cellular aging markers (TL and mtDNA copy number); these secondary outcomes were also assessed at the time of EndoPAT measurement.

The primary outcome studied was the reactive hyperemia index (RHI, a measure of endothelial function), as both a categorical and continuous outcome. Continuous RHI was standardized by dividing by the SD to make results interpretable as change per unit of SD. Peripheral arterial tonometry (PAT) is a measure of both macrovascular and microvascular peripheral function. The EndoPAT device uses a finger probe to assess digital volume changes accompanying pulse-waves. Baseline measurements are performed for 6 minutes, followed by a 5-minute occlusion phase via inflation of a blood pressure cuff. Following occlusion, PAT signal is recorded for 5 minutes and the signal analyzed by computer algorithm. The device calculates RHI, reflecting peripheral vascular function.

RHI is postocclusion-to-preocclusion PAT signal ratio in the occluded side, normalized to the control side, and further corrected for baseline vascular tone, with normal: RHI greater than 1.67 and abnormal: RHI of 1.67 or less based on prior studies (19, 20). RHI has a sensitivity of 85% and specificity of 80% for detecting endothelial dysfunction in coronary artery disease patients (19, 20). A strict quality-controlled protocol was followed to reduce random variability: The procedure was completed in a quiet, temperature-controlled room, fasting for at least 8 hours; blood pressure was measured at least 20 minutes prior to EndoPAT administration, and fasting serum laboratory samples were drawn after completion. RHI was evaluated as both a categorical and continuous variable.

Secondary outcomes included the following: two measures of CVD risk (AHA PREVENT score, metabolic syndrome), and two measures of cellular aging (TL, mtDNA). The PREVENT score is a measure of 30-year atherosclerotic CVD (ASCVD) risk taking into account traditional risk factors (smoking status, cholesterol, systolic blood pressure, antihypertensive or statin use, and diabetes) and estimated glomerular filtration rate. Models were sex specific, race free, and developed on age scale and adjusted for competing risk of non-CVD death (29). Metabolic syndrome is defined as meeting 3 out of the following 5 components: waist circumference of 88 cm or greater, triglycerides of 150 mg/dL or greater, high-density lipoprotein less than 50 mg/dL, elevated blood pressure (systolic ≥130 mm Hg or diastolic ≥85 mm Hg) or hypertensive drug treatment, fasting glucose of 100 mg/dL or greater (30). Primary and secondary outcomes were chosen based on lack of literature regarding these outcomes relating to ovarian aging in a longitudinal format, as well as data availability in OVA and a desire to evaluate potential common underlying pathology associated with CV risk.

### Cellular aging assessment

#### Telomere length

Peripheral blood mononuclear cells were purified from whole blood collected in Vacutainer CPT Tube with Sodium Citrate (BD, catalog No. 362761) and cells were stored as dry pellets at −80 °C and total genomic DNA was purified using the QIAamp DNA Mini kit (QIAGEN; catalog No. 51104) and stored at −80 °C for batch TL measurement. The TL assay is adapted from the published original method by Cawthon (31). Details of the protocol can be found at the Telomere Research Network's website (https://trn.tulane.edu/wp-content/uploads/sites/72/2021/07/Lin-qPCR-protocol-01072020.pdf) and in Appendix A) (32). The assay coefficient variation is 2.6% ± 2.0%. Intraclass correlation of repeat DNA extraction (N = 48) is *R* = 0.913 (95% CI, 0.85-0.95).

#### Mitochondrial DNA copy number

Detection of a 69-bp fragment of the ND1gene in mtDNA (nucleotides 3485-3553) and an 87-bp fragment of RNase P (TaqMan Copy Number Reference Assay, human, RNase P, catalog No. 4403328, Life Technologies) by TaqMan assays is used to determine the relative copy number of mtDNA per diploid nuclear genome as previously described (33, 34). The average interassay coefficient of variation is 2.3 ± 1.5%. The same DNA samples used for TL assay were used for mtDNA.

### Statistical analysis and covariates

To account for common predictors of ovarian reserve and CV risk, our study used 2 statistical models. Model 1 included covariates related both to the predictor and outcome, while model 2 was a larger model that also included covariates related to the outcome only. Accordingly, 2 lists of variables were selected a priori: (1) covariates that can affect both the predictor (ovarian reserve) and outcome (CV risk), and (2) covariates that can affect only the outcome of interest. The covariates for model 1 include age (both at enrollment and EndoPAT), race, body mass index (BMI), smoking, and socioeconomic markers (income and education). The covariates for model 2 included all covariates in model 1 as well as additional variables: hyperlipidemia, hypertension, homeostatic model assessment of insulin resistance (HOMA-IR), and CRP.

**Figure 2:**
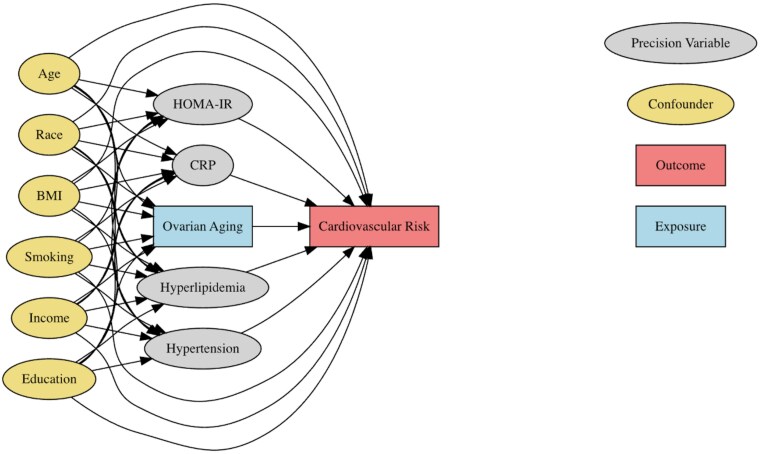
Directed acyclic graph (DAG) showing variables (confounders, mediators, effect modifiers, and precision variables). Model 1 variables are assumed to be confounders, and Model 2 variables are considered precision variables (affected by confounders, related to the outcome, but not on the causal pathway).

Due to potential common confounders affecting both the outcome and predictors of interest, analysis was performed to examine the association between ovarian reserve markers and the covariates in model 1 to investigate associations that may affect the pathway between the predictor, confounders, and outcome. Fig. 2 (directed acyclic graph) illustrates the relationships between these covariates. Two models were constructed: “adjusted,” which included the variables in (1), and “fully adjusted,” which included the variables in (1) and (2), respectively. The main results focused on the fully adjusted model given the inclusion of parameters that can affect both endothelial function and/or ovarian aging.

For the primary and secondary outcomes, logistic and linear regression were used to study categorical and continuous outcomes, respectively, in relation to baseline ovarian reserve measures, with adjustment for covariates as described earlier. The primary outcome of RHI was studied as both a categorical(with a cutoff for normal of 1.67 for the categorial variable as described previously) (19, 20) and as a continuous variable, while cellular aging markers were studied as only continuous outcomes. AHA PREVENT 30-year risk scores were modeled as continuous outcomes in linear regression analyses. Additionally, AMH and AFC were categorized into quartiles to facilitate clinical interpretability and presentation of effect estimates. Adjusted mean 30-year ASCVD risk (%) and 95% CIs were estimated from model-based marginal predictions, averaging over the observed covariate distributions. Metabolic syndrome was studied as only a categorical outcome (yes/no based on meeting at least 3/5 criteria). A mediation analysis using linear regression was also performed that examined the relationship of ovarian reserve measures AMH and AFC with the variables in model 1 to examine possible direct or indirect effects on predictors.

All participants were premenopausal at baseline. A sensitivity analysis was also performed, for the primary outcome (RHI), using only those remaining premenopausal at the time of EndoPAT measurements, as menopause and associated drop in estradiol levels increases CV risk profile. All tests were 2-sided with a statistical significance at the α = .05 level. Data analysis was performed using STATA version 16 (StataCorp). Results reporting adhered to the STROBE guidelines (35).

## Results

The cohort included 322 OVA participants (out of 829 total, 38.8%) with EndoPAT RHI measurement available. This cohort was average 35.4 years (SD 5.6) at the time of the OVA study enrollment (similar to overall cohort, which was 35.2 SD 5.5 years at baseline), and 45.1 years (SD 6.0) at the time of EndoPAT measurement consistent with designed enrollment (Table 1). The cohort was 27.0% Asian-Chinese (87/322), 18.9% Black (61/322), 24.5% Hispanic (79/322), and 29.5% White (95/322). The mean BMI was in the overweight range at 28.7, SD 7.4), and the majority were nonsmokers. A small percentage of the cohort had diabetes at baseline (5.6%-18/322), corresponding with overall normal average fasting insulin, glucose, and HOMA-IR values. Hypertension and metabolic syndrome were both present in roughly 20% of the cohort (62/322 for hypertension and 63/322 for metabolic syndrome), and hyperlipidemia (total cholesterol >200) was present in nearly half the participants at OVA baseline.

**Table 1:** OVA.CV characteristics for the Endothelial and Peripheral Arterial Tone study

Characteristic	Subcategory	OVA.CV cohort (322)	Excluded (758)	*P*
Age at baseline (OVA.CV enrollment), y		35.0 (5.6)	34.5 (5.5)	.177
Mean (SD)				
Race/Ethnicity		No. (%)		
	Asian	87 (27.0)	210 (27.7)	.075
	Black	61 (18.9)	192 (25.3)	
	Hispanic	79 (24.5)	175 (23.1)	
	White	95 (29.5)	181 (23.9)	
BMI		27.0 (7.2)	27.2 (6.9)	.791
Smoking		No. (%)		
	No	251 (78.0)	584 (77.2)	.773
	Yes	71 (22.1)	173 (22.9)	
Income household, $		No. (%)		
	<50 000	139 (43.4)	351 (46.6)	.651
	50 000-99 999	117 (36.6)	266 (35.3)	
	≥100 000	64 (20.0)	137 (18.2)	
Education	<HS	24 (7.5)	66 (8.7)	.254
	Some HS/HS grad	26 (8.1)	89 (11.7)	
	Some college	85 (26.5)	182 (24.0)	
	College degree	112 (34.9)	274 (36.2)	
	Graduate/Prof degree	74 (23.1)	147 (19.4)	
				
AMH at baseline, ng/mL		4.4 (3.7)	4.7 (4.1)	.309
Mean (SD)				
AFC at baseline		15.3 (9.7)	15.2 (9.6)	.875
Mean (SD)				
Fasting glucose		86.6 (8.3)	87.0 (8.5)	.466
Mean (SD)				
HOMA-IR		1.7 (2.3)	1.6 (1.4)	.404
Mean (SD)				
CRP		3.2 (4.3)	4.0 (5.9)	.060
Mean (SD)				
mtDNA copy No.		618.8 (184.1)	632.9 (208.4)	.384
Mean (SD)				
Telomere T/S ratio		1.01 (0.17)	0.96 (0.16)	<.001
Mean (SD)				

Abbreviations: AFC, antral follicle count; AMH, antimüllerian hormone; BMI, body mass index; CRP, C-reactive protein; HOMA-IR, homeostatic model assessment of insulin resistance; HS, high school; mtDNA, mitochondrial DNA; OVA, Original Ovarian Aging Cohort; OVA.CV, follow-up cohort assessing cardiovascular risk; T/S, telomeric DNA (T) to the β-globin single copy gene (S), calibrated to a plate reference genomic DNA sample.

At baseline (time 1), the average AMH value was 4.4 ng/mL and the average AFC was 15.1 (SD 9.7). At the time of EndoPAT (time 2), the AMH value had dropped to 2.3 (SD 2.9); the relatively high AMH for age was driven by outlier high values among some of the younger patients in the cohort. The average EndoPAT RHI score was 2.3 (SD 0.7), which is in the normal range, and the 30-year AHA PREVENT risk score at time 2 was 11.7% (SD 7.6%). In this cohort, 66 of 322 (20.5%) of participants had abnormal RHI (≤1.67) values.

RHI as a continuous outcome was significantly associated with both increased AMH and AFC on fully adjusted models (AMH coefficient 0.052; 95% CI, 0.008-0.096; *P* = .02; AFC coefficient 0.017; 95% CI, 0.0013-0.032; *P* = .04). For this continuous outcome, there were no statistically significant associations with AMH or AFC in unadjusted or minimally adjusted models controlling for age, race, BMI, smoking, and socioeconomic markers. For RHI as a categorical outcome, there were no statistically significant associations in any of the models with AMH or AFC (Table 2).

**Table 2:** Primary outcome: reactive hyperemia index

	RHI categorical		RHI continuous	
	OR (95% CI)	*P*	Coeff (95% CI)	*P*
AMH no adjustment	1.03 (0.95 to 1.12)	.47	0.019 (−0.023 to 0.046)	.50
AMH adjusted*^a^*	1.07 (0.96 to 1.20	.22	0.033 (−0.009 to 0.075)	.12
AMH fully adjusted*^b^*	1.10 (0.98 to 1.24)	.12	0.052 (0.008 to 0.096)	.02
AFC no adjustment	1.01 (0.98 to 1.04)	.48	−0.001 (−0.013 to 0.011)	.89
AFC adjusted*^a^*	1.02 (0.99 to 1.06)	.22	0.008 (−0.008 to 0.022)	.29
AFC fully adjusted*^b^*	1.04 (0.99 to 1.08)	.09	0.017 (0.001 to 0.032)	.04

RHI values normalized by SD. RHI categorical: normal greater than 1.67. AMH and AFC as primary predictors.

Abbreviations: AFC, antral follicle count; AMH, antimüllerian hormone; BMI, body mass index; Coeff, coefficient; CRP, C-reactive protein; EndoPAT, Endothelial and Peripheral Arterial Tone; HOMA-IR, homeostatic model assessment of insulin resistance; OR, odds ratio; RHI, reactive hyperemia index.

^*a*^Adjusted model covariates: age (at enrollment and EndoPAT), race, BMI, smoking, income, and education.

^*b*^Fully adjusted: age (at enrollment and EndoPAT), race, BMI, smoking, income, education, hyperlipidemia, hypertension, HOMA-IR, and CRP.

For the secondary outcomes including metabolic and cellular aging (Table 3), the only statistically significant association was seen in the fully adjusted model (model 2) for AFC with metabolic syndrome (odds ratio [OR] 0.92; 95% CI, 0.86-0.99; *P* = .02). No other associations of AMH or AFC with any secondary outcomes were statistically significant.

**Table 3:** Secondary outcomes

	AHA 30-y ASCVD		Metabolic syndrome	
	Coeff (95% CI)	*P*	OR (95% CI)	*P*
AMH not adjusted	−0.074 (−0.104 to –0.44)	<.001	0.89 (0.80 to 0.98)	.02
AMH adjusted*^a^*	−0.004 (−0.031 to 0.023)	.766	0.89 (0.76 to 1.05)	.16
AMH fully adjusted*^b^*	−0.015 (−0.044 to 0.014)	.308	0.85 (0.70 to 1.03)	.10
				
AFC not adjusted	−0.024 (−0.036 to −0.012)	<.001	0.99 (0.95 to 1.02)	.31
AFC adjusted*^a^*	0.004 (−0.007 to 0.014)	.517	0.98 (0.94 to 1.03)	.41
AFC fully adjusted*^b^*	−0.006 (−0.018 to 0.005)	.273	0.92 (0.86 to 0.99)	.02

	mtDNA		TL	
	Coeff (95% CI)	*P*	Coeff (95% CI)	*P*
AMH not adjusted	6.00 (−4.12 to 16.13)	.24	0.01 (0.0033 to 0.017)	.004
AMH adjusted*^a^*	4.74 (−7.87 to 17.35)	.46	0.001 (−0.007 to 0.010)	.68
AMH fully adjusted*^b^*	4.56 (−9.04 to 18.15)	.51	0.000 (−0.009 to 0.010)	.92
AFC not adjusted	2.74 (−0.52 to 6.00)	.10	0.004 (0.001 to 0.006)	.002
AFC adjusted*^a^*	1.80 (−2.24 to 5.85)	.38	0.001 (−0.001 to 0.004)	.30
AFC fully adjusted*^b^*	3.09 (−1.42 to 7.61)	.18	0.001 (−0.003 to 0.004)	.44

Abbreviations: AFC, antral follicle count; AHA, American Heart Association; AMH, antimüllerian hormone; ASCVD, atherosclerotic cardiovascular disease; BMI, body mass index; Coeff, coefficient; CRP, C-reactive protein; EndoPAT, Endothelial and Peripheral Arterial Tone; HOMA-IR, homeostatic model assessment of insulin resistance; mtDNA, mitochondrial DNA; OR, odds ratio; TL, telomere length.

^*a*^Adjusted model covariates: age (at enrollment and EndoPAT), race, BMI, smoking, income, and education.

^*b*^Fully adjusted: age (at enrollment and EndoPAT), race, BMI, smoking, income, education, hyperlipidemia, hypertension, HOMA-IR, and CRP.

In the mediation analysis, AMH was significantly associated with age BMI, at time 1 and 2, but no other factors; this suggests that BMI may have an indirect effect on the outcome of interest. AFC was significantly affected only by age at baseline. Lastly, as a sensitivity analysis, the same AMH and AFC associations with primary and secondary outcomes were investigated in only the premenopausal cohort (N = 246), given a possibly different CV risk profile in this population (Supplementary Tables S1 and S2) (32). The positive association with continuous RHI and AMH was still present (coefficient 0.050; 95% CI, 0.003-0.096; *P* = .04) in the fully adjusted model. AFC had a similar coefficient (0.016 vs 0.017) in relation to continuous RHI in the adjusted analysis, though the *P* value was no longer statistically significant .092. Similar to the main analysis, no other significant associations were found with categorical RHI or in any of the other primary outcome models. For secondary outcomes, no significant associations with AMH or AFC were present in any of the adjusted models. For easier interpretability, AHA PREVENT effect sizes across AMH and AFC quartiles are shown in Supplementary Table S3 (32).

## Discussion

In summary, in the prospectively recruited OVA cohort, higher baseline ovarian reserve (AMH/AFC) was positively associated with endothelial function after adjustment for covariates. However, baseline ovarian reserve markers had no significant association with metabolic or cellular aging measurements after adjustment for covariates (with the exception of higher AFC associated with lower odds of metabolic syndrome). A sensitivity analysis of the premenopausal cohort yielded similar results, though endothelial dysfunction as a continuous variable was associated with AMH only, likely in part due to smaller sample size.

### Comparison to existing literature

The relationship between ovarian reserve and CV risk has not been well established for women in the absence of ovarian failure or polycystic ovary syndrome (PCOS), though limited studies suggest early diminished ovarian reserve (DOR) may be associated with increased CV risk. This association has some biological plausibility; DOR in some studies was a proxy for POI, which is linked with CV risk through mechanisms including decreased estradiol levels. Several studies have also reported women with DOR may have increased CVD risk markers including elevated HOMA-IR, CRP, and lipid markers (3, 5). However, the definition of DOR used in literature can be heterogeneous, ranging from POI to slightly diminished AMH. This limits the generalizability of these findings and the ability to understand the role of estrogen decline in risk. There is a particular dearth of literature on the relationship between CV risk and ovarian aging during the reproductive years when ovarian reserve, and estrogen levels, are within normal limits, which this study aims to investigate.

To summarize existing literature on the subject, the Doetinchem Cohort reported among 3108 female participants aged 20 to 60 years, that each additional ng/mL/year decrease of log AMH was associated with significantly higher CVD (hazard ratio 1.46) and coronary heart disease (hazard ratio 1.56) incidence. Endothelial function was not studied in this cohort (6). Another study of 70 premenopausal women with normal ovulation (aged 32.7 ± 6.5 years) reported AMH concentration was negatively associated with subclinical atherosclerosis indices, even when controlling for established CV risk factors (7). Several studies have also investigated CVD risk and AMH levels in men, including a prospective cohort study of 394 men (aged 40-80 years) that found that higher baseline AMH levels were associated with lower carotid intima-media thickness at baseline and lower mean plaque scores at follow-up (35, 36). Another study found that among 989 men with median follow-up 9.4 years, there was an independent and inverse association between AMH and all-cause mortality. While these studies suggest an inverse association between gonadal reserve measures and CVD risk, the relationship specifically between endothelial function and ovarian reserve, the subject of this study, has not been widely investigated.

This study is unique in studying the relationship between ovarian reserve and endothelial function in reproductive-age women with a longitudinal design. Despite restricting participants to those with regular cycles (excluding PCOS and POI), this study still found (similar to prior studies) decreasing levels of ovarian reserve may be associated with increased CVD risk. In particular, this relationship was found when studying RHI score as a continuous but not categorical outcome, suggesting, in this relatively young population, a binary threshold may not be clinically meaningful. Additionally, this study's relatively young cohort has a low risk of CV events, which also may have implications on the clinical significance of the findings; additional study is warranted as this cohort ages, as it is possible that increased clinical significance may be seen with aging. The decrease in estradiol associated with menopause or POI is known to increase CV risk (37-39). However, physiologic estradiol levels do not have significant variation in women with regular cycles and normal ovarian reserve (40, 41), so differences in endothelial function or CV risk in this population occurs via other mechanisms which may include inflammatory or cellular aging mechanisms; this area is not well understood and warrants further investigation.

To investigate potential common underlying risk both for ovarian aging and CV risk, this study examined secondary outcomes as a potential source of the findings that could not be explained by lowered estradiol and classic ovarian aging. Existing literature on cellular aging and ovarian reserve include the population-based CARDIA cohort, which reported that AMH (collected at an average of 40.2 years), had no association with subsequent measures of cellular aging markers of telomere length, mtDNA copy number, or epigenetic age acceleration (11). Similarly, our analysis also did not find relationships between ovarian reserve and markers of cellular aging. However, in prior studies, the baseline AMH was measured at an older age when variation is less (42). Several other studies have reported women with POI have decreases in TL (12, 13) and decreased mtDNA copy number (14, 15) compared to women with normal ovarian function, though these studies cannot necessarily be extrapolated to levels in women with normal ovarian reserve. It is also possible changes are occurring locally at the ovary and not evident in systemic measurement. Prior studies have found a possible linkage between ovarian reserve and metabolic risk, including a prior OVA study of 951 women that found the number of cardiometabolic risk factors was 52.1% higher among participants with low compared to high AMH, though associations were attenuated when BMI was covaried (16). Another study of 136 individuals found ovarian volume was lower in women with metabolic syndrome compared to healthy control individuals, though AFC did not significantly vary between groups (43). Our study also found AFC was associated with lower metabolic risk after full adjustment, though most other secondary outcomes were not statistically significant. The wide CIs in this study, potentially due to sample size, cannot rule out important associations, even though they were not statistically significant. More detailed evaluation in this area is warranted to understand the relationship between ovarian aging and somatic markers of aging and metabolic dysfunction.

Overall, primary study findings are in agreement with some prior studies as described earlier. In these young women without clinical ovarian dysfunction syndromes, AMH and AFC predict CVD risk factors (adjustment variables in model 2), plus beneficially and independently also influence endothelial function in terms of RHI score. This contributes to the literature with its novelty, well-established prospective cohort, and investigation of robust primary and secondary outcomes with control of covariates. Additionally, this study is a prospective longitudinal collection of a racially and ethnically diverse population, which is rare in the literature and adds additional validity, and generalizability, to the findings. These findings suggest ovarian reserve (even within a normal range) may have associations with subsequent endothelial dysfunction through mechanisms other than lowered estrogen and cellular aging and may become an early marker of a population at risk. Given the importance of vascularity for later maturation of the ovarian follicle (those assessed in AFC and AMH), another opportunity for exploration would be to study vascularity within the ovary and correlation with CV markers and systemic vascular changes with aging.

### Strengths

The strengths of this study include the prospectively recruited, multiethnic, OVA cohort, which allowed collection of longitudinal data on ovarian reserve and endothelial health, with an average of more than 9 years between measurements. This study is also strengthened by its novelty; as described earlier, the literature on normal ovarian reserve in relation to CV risk and cellular aging markers is very limited. In addition, the EndoPAT device is a reliable, noninvasive, and clinically validated device for measuring endothelial dysfunction. Measurements of ovarian reserve were collected using transvaginal ultrasound with well-trained clinicians and standardized AMH assays. This study also had access to robust secondary outcomes, including markers of cellular aging and metabolic risk. The cohort was a community-based multiethnic cohort, increasing the generalizability of the findings; OVA is one of the largest and most diverse study populations to date, as many community-based cohorts are of majority White ethnicity. OVA also has access to extensive confounder data, which enabled the investigation of multiple outcomes with adjustment. Lastly, the sample size was fairly robust for the detailed outcomes studied.

### Limitations

This study has several limitations. The OVA study was focused on reproductive-age participants with regular cycles. This excluded participants with POI and PCOS, which may limit the generalizability of the findings to the extremes of ovarian function, particularly given the heterogeneity of diminished ovarian reserve definitions in the literature. OVA participants were also recruited exclusively in the San Francisco Bay Area, which may not be entirely a generalizable population to other geographic locations. In addition, only one RHI measurement was used, as there were no data available on longitudinal endothelial function data. Related to this, the age of the cohort was relatively young; having access to endothelial function data at older ages may be more informative as CV risk and events become much more prevalent with increasing age and time after menopause. It is possible differences in CV risk may not be captured at younger ages, or that such differences are attenuated. Due to the young age of the cohort, we used the 30-year AHA PREVENT equation to calculate risk. Despite this, no association was noted between baseline assessment of ovarian aging and long-term prediction calculations. It is important to understand the EndoPAT and the epidemiologic prediction scores evaluate different factors and may not agree, as seen in this study. Subclinical measures, such as EndoPAT, look at a pathophysiological measure, while prediction tools are statistical estimates. A combination may yield the most reliable information and should be further studied. In addition, having ovarian reserve measurements from several standardized time points would have been useful in terms of increasing the rigor of the study and the generalizability of the findings. Additionally, the assumption has been made that AFC and AMH are reflective of true ovarian reserve (primordial follicle pool) while these are more accurately a reflection of the antral follicles, and the antral + preantral pool, respectively. Lastly, there was also no information on actual CV events (given the young age of the participants), instead focusing on surrogate markers of CV risk.

### Conclusions

In this longitudinal cohort of women with normal ovarian aging and regular menstrual cycles, baseline ovarian reserve markers of AMH and AFC were positively associated with endothelial function as a continuous outcome. However, baseline ovarian reserve markers were not related to endothelial function as categorical outcome or to most secondary outcomes of cellular aging and metabolic risk. Future studies will investigate the effect of rate of decline in AFC and AMH on these relationships and evaluate older populations and across multiple time points. The incidence of actual CV events, and studies to elucidate the mechanisms underlying these complex relationships, remain for future studies.

## Data Availability

Original data generated and analyzed during this study are included in this published article or in the data repositories listed in “References.”

## Abbreviations

AFC: antral follicle count
AHA: American Heart Association
AMH: antimüllerian hormone
ASCVD: atherosclerotic cardiovascular disease
BMI: body mass index
CRP: C-reactive protein
CVD: cardiovascular disease
DOR: diminished ovarian reserve
EndoPAT: Endothelial and Peripheral Arterial Tone
HOMA-IR: homeostatic model assessment of insulin resistance
mtDNA: mitochondrial DNA
OR: odds ratio
OVA: Ovarian Aging Study
PAT: peripheral arterial tonometry
PCOS: polycystic ovary syndrome
POI: premature ovarian insufficiency
RHI: reactive hyperemia index
RH-PAT: reactive hyperemia peripheral arterial tonometry
TL: telomere length
